# Spatiotemporal role of muscarinic signaling in early chick development: exposure to cholinomimetic agents by a mathematical model

**DOI:** 10.1007/s10565-022-09770-w

**Published:** 2022-09-13

**Authors:** Ombretta Paladino, Arianna Moranda, Carla Falugi

**Affiliations:** 1 Department of Civil, Chemical and Environmental Engineering (DICCA), University of Genoa, Via Opera Pia 15, 16145 Genoa, Italy; 2 Department of Earth, Environmental and Life Sciences (DISTAV), University of Genoa, Corso Europa 26, 16132 Genoa, Italy

**Keywords:** Chick early development, Cholinergic molecules, Muscarinic receptors, Exposure modeling, Inductive messages, Interference mechanisms

## Abstract

Awareness is growing that, besides several neurotoxic effects, cholinomimetic drugs able to interfere the cholinergic neurotransmitter system may exert a teratogen effect in developing embryos of vertebrate and invertebrate organisms. Cholinomimetic substances exert their toxic activity on organisms as they inhibit the functionality of the cholinergic system by completely or partially replacing the ACh molecule both at the level of the AChE active site and at the level of acetylcholine receptors. In this work, we focused the attention on the effects of muscarinic antagonist (atropine) and agonist (carbachol) drugs during the early development and ontogenesis of chick embryos. An unsteady-state mathematical model of the drug release and fate was developed, to synchronize exposure to a gradient of drug concentrations with the different developmental events. Since concentration measures in time and space cannot be taken without damaging the embryo itself, the diffusion model was the only way to establish at each time-step the exact concentration of drug at the different points of the embryo body (considered two-dimensional up to the 50 h stage). This concentration depends on the distance and position of the embryo with respect to the releasing source. The exposure to carbachol generally enhanced dimensions and stages of the embryos, while atropine mainly caused delay in development and small size of the embryos. Both the drugs were able to cause developmental anomalies, depending on the moment of development, in a time- and dose-dependent way, regardless the expression of genes driving each event.

**Graphical abstract:**

1. Early chick embryos were exposed to muscarinic drugs in a spatial-temporal context.
2. Effects were stage-(time) dependent, according to distance and position of the source.
3. Atropine inhibited growth, mainly interfering with the cephalic process formation and heart differentiation; carbachol increased growth reducing differentiation.
4. Interferences may be exerted by alteration of calcium responses to naturally occurring morphogen-driven mechanisms.

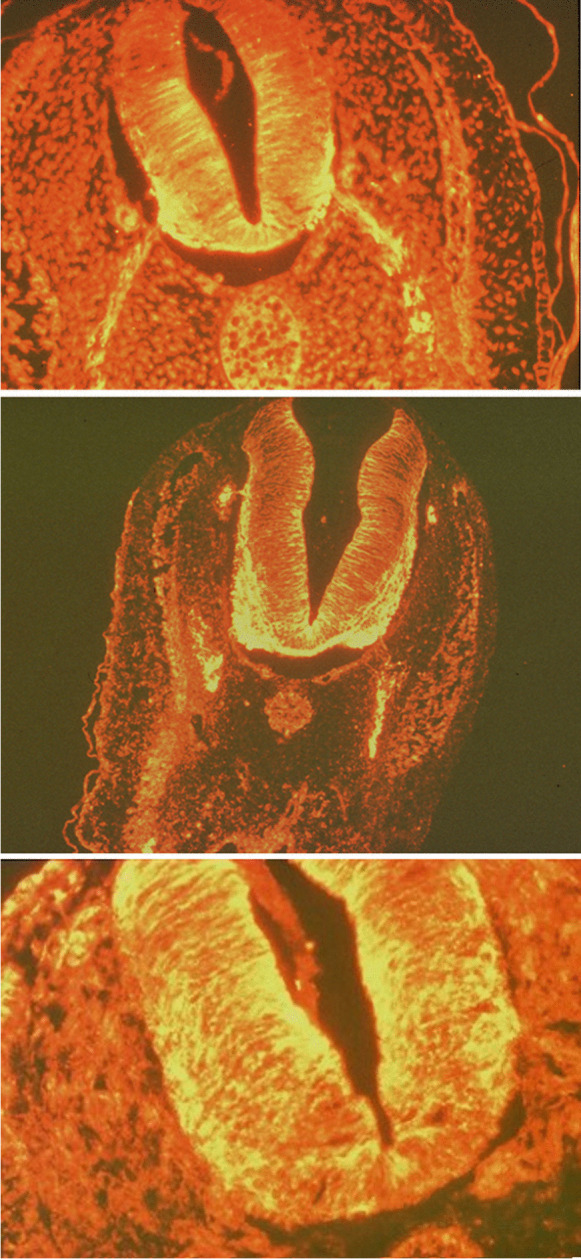

**Supplementary Information:**

The online version contains supplementary material available at 10.1007/s10565-022-09770-w.

## Introduction

The presence and possible role of molecules related to neurotransmitter systems in non-neuromuscular and pre-nervous tissues and cells has been reported since long time (Buznikov et al. 1968; 1996; Buznikov and Shmukler 1981), and the embryonic role of the cholinergic system has been demonstrated by different researchers (Wessler and Kirkpatrick 2008; Falugi and Aluigi 2012, for reviews). In this light, the teratogenic effect of cholinomimetic pesticides is known since the half of the last century (Misawa et al. 1982), and it is matter of regulation by several governments (e.g., EU Regulation n. 1107/2005, 2009, Directive 2009/128/EC). Cholinomimetic substances exert their toxic activity on organisms as they inhibit the functionality of the cholinergic system by completely or partially replacing the ACh molecule both at the level of the AChE active site (Sultatos 1994) and at the level of acetylcholine receptors (AChRs, Bakry et al. 1988). Embryotoxicity may be due to interference in inductive message exchanges mediated by ion fluxes and intracellular ionic concentration change. Here, we focused the attention on the developmental function of muscarinic acetylcholine receptors (mAChRs), which are responsible for the modulation of intracellular Ca^2+^ concentration (Berridge 1981; 1986). The role of mAChRs in development and differentiation has been mainly studied in the vertebrate’s eye (Angelini et al. 1998) and for its role in regulating the visual function (Groleau et al. 2015). Our thesis is that mAChRs may be involved in the regulation of the correct body patterning of vertebrates, by interacting, in normal conditions, with the reception of positional information along the different phases of development. MAChRs blockade/activation has been here investigated using the cholinomimetic antagonist and agonist drugs atropine (AT) and carbamylcholine (CCh) respectively, which are non-selective among the different muscarinic AChR forms (mAChRs). The experiments have been carried out by regulating the exposure in a space-temporal pattern synchronic with the single developmental events, along the cephalic-caudal morphogenesis, primary induction, and specification of the morphogenetic fields up to the stage of 50 h incubation. The responses of the chick embryos were analyzed by using an analytical solution of the diffusion equation, able to compute the concentration in time of the cholinomimetic drugs, i.e., where and when cell and tissue interactions are responsible for positional information exchange.

## Materials and methods

The chemicals were obtained from Sigma Chem Co., unless otherwise stated. As a vertebrate model, the early development of chick embryos has been chosen. Early stages do not present bioethical concerns, as up to stage 15 HH, the neural crest is not yet differentiated to form sensorial ganglia and sense nerves. Fertilized eggs were obtained by a commercial hatchery (Ladi hatchery, Carasco, GE, Italy), brought to the laboratory and immediately put in incubator at 38.5 °C.

### Cholinomimetic substances and inhibitors

*Atropine (AT)*. Competitive nonselective antagonist at central and peripheral muscarinic acetylcholine receptors. A0132 Sigma-Aldrich; purity ≥ 99% (TLC), powder, water solubility: 2 mg/mL. Molecular weight 289.37; EC Number 200–104-8. Mother solution is stable for several days at 4 °C.

*Carbamylcholine (Carbachol, CCh)*. Non-selective cholinergic agonist (analog of acetylcholine) that is resistant to the action of cholinesterases. C4382 Sigma-Aldrich, purity grade ≥ 98% (titration), crystalline. Molecular Weight 182.65; EC Number 200–127-3. Water solubility 1 g/ml.

### Experimental procedure

For each experiment, 3 groups of 60 eggs were employed. The experiments were repeated at different seasonal times. The eggs were incubated flat and unturning before the treatment.

Some previous experiments were made by injection of the drugs into the sub germinal chamber to establish the final drug concentrations suitable to be used in this work.

Three microliters of different concentrations of the drugs, in Tyrode solution (TS, Tyrode 1910), or bovine serum albumin (BSA), was injected in the sub germinal chamber, using a Hamilton syringe. The concentration able to cause effects on each developmental event (LOEC) was identified as 10^−4^ M for AT and 10^−3^ M for CCh.

#### Staging of embryos

Control embryos were staged according to the incubation time and the morphology, as described by HH (Hamburger and Hamilton 1951) and by the number of somite pairs (Fig. 8 in Appendix).

The treated embryos were staged according to the incubation time and compared with the control embryos incubated for the same time.

#### Exposure to the cholinomimetic drugs and spatial–temporal distribution

Fertilized eggs of white leghorn chicken were incubated at 38.5 °C in a controlled humidity incubator with 12 h circadian rhythm, for 20 and 24 h to obtain embryos at stages 4 and 6, respectively. The eggs were taken from the incubator and opened under a stereomicroscope in sterile condition, under a laminar flux hood. A window of about 1 cm diameter was made in the eggshell, corresponding to the position of the blastodisc, to reach the embryo surface.

A piece of agar, soaked with the test substances, was posed over the vitelline membrane, which is freely permeable to several substances dissolved in water, (Pons et al. 1985; Garcia et al. 1983; Rymen and Stockx 1974).

**Fig. 1:**
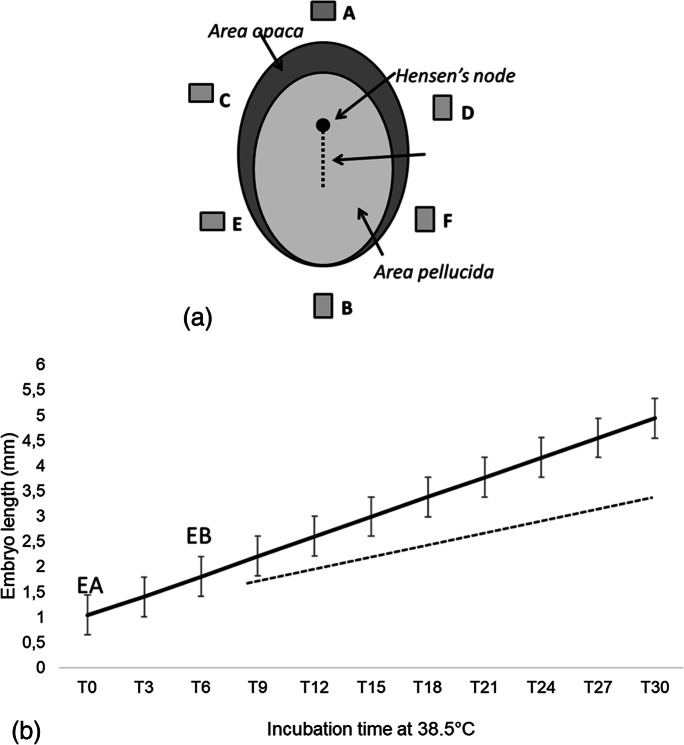
Estimation of the position of embryo structures. **a** Scheme of a stage 4 HH, corresponding to time 0 (T0) of the experiment, the rectangles show the positions of the agar: (A) cephalic, (B) caudal; (C, D) lateral cephalic; (E, F) lateral caudal. **b** Theoretical growth of the control embryos during the experimental procedure: EA, exposure since 20 h incubation, EB, exposure since 24 h. The dotted line suggests the position of heart. This graph was used to extrapolate retrospectively the position of the different morphogenetic fields along time, to be compared with drugs distribution in space and time

The agar was prepared by dissolving 20 g of Bacto Agar in 1 L TS, without stirring, in a thermostatic bath at 80 °C. After melting, the liquid was poured in a petri dish up to 1 mm high. After cooling and solidification, a parallelepiped of 2 × 2 × 3 mm was cut by a microscalpel (Moria, Paris) and soaked in 3 μL of 1 mM AT/TS or 2 μL of 0.1 M carbachol/BSA. The piece of agar was placed on the vitelline membrane at different distances from the opaque area. The position was cephalic or lateral or caudal to the embryo (see Fig. 1a). For controls, agar was soaked with sterile TS. With a pipette, the albumen was drawn to remove the liquid and free the surface of the membrane, so that the piece of agar could stick stably, then the shell was again filled with 1/9 albumen/TS, supplemented with 1% glucose. To maintain synchronic development, the eggs were taken simultaneously from the incubator, maintained outside along all the procedure (lasting some hours for 30 eggs), and put again into the incubator at the same time.

After treatment, the egg window was sealed with the removed shell fragment, held on a piece of UV sterile cello-tape. The eggs were posed in the incubator and maintained unturned up to 33 and 50 h total incubation, respectively.

After the incubation, control and treated embryos were collected and rinsed in TS and the solution was drawn, till the embryos remained flat at the bottom of the Petri dish. Then, pre-cooled 3% paraformaldehyde (PFA) in TS was added drop by drop to cover the embryos. Fixation lasted 45 min at 4 °C, then the embryos were rinsed in different buffers, according to the subsequent procedures: in 0.1 M maleate buffer pH 6.0 for AChE activity localization; in PBS pH 7.4 for immunohistochemical staining. Staging of the embryos was made according to HH, the stage of exposed embryos was referred to the stage of the respective unexposed control embryos. The embryos stained for the revelation of AChE activity, were then dehydrated, mounted in resin, and observed under a Leitz microscope, equipped with a micrometric objective, to measure the embryos and control the distance from the agar.

### Morphological and biochemical analyses

#### Histochemical localization of AChE activity

Embryos, fixed in 2% PFA/TS for 15 min at 4 °C, were rinsed in 100 mM maleate buffer pH 6.0 and incubated in the medium suggested by Karnovsky and Roots (1964), containing 100 mM Na citrate, 30 mM Cu^++^ sulfate and 100 mM K^+^ ferricyanide in 100 mM maleate buffer, pH 6.0, 10 mg acetylthiocholine iodide (AcTChI, specifically hydrolyzed by AChE, and, with minor efficiency, by butyrylcholinesterase (BChE). The incubation was carried out in the dark at 4 °C overnight. Controls for the specificity of the reaction were performed by incubation without substrate or by pre-incubating for 30 min in a medium containing 1 μM eserine (Physostigmine), non-selective inhibitor of cholinesterases, of BW284c51 (anti-AChE, Burrough-Wellcome, USA).

#### Biochemical measurement

AChE activity was measured by the quantitative method of Ellman et al. (1961), which was modified ad hoc for the Jenway spectrophotometer (6405 Jenway, Gransmore Green, UK).

Ten microliters of 0.5% Triton X-100 extracts was incubated in the presence of 50 μL of the substrates acetyl-β-metyl thiocholine iodide (AcMTChI) or butyrylthiocholine iodide (BuTChI) in phosphate buffer, pH 8.0, and stained by 50 μL of dithiobis-nitrobenzoic acid (DTNB). The reaction was allowed to develop for 10 min at room temperature and the absorption was measured at 412 nm and compared to a blank obtained by omitting the substrate. The enzyme activity was expressed in units = μmol of ACh hydrolyzed min^−1^ mg_protein_^−1^. Each measurement was performed in triplicate and related to total protein content (Bradford protein assay, measured at 595 nm wavelength on the same spectrophotometer).

#### Immunohistochemical localization of α-tubulins

α-Tubulin labeling was used to detect the neurofilaments (Breuss et al. 2017) emerging from neural tube cells.

After fixation, the embryos were dehydrated with alcohol, clarified in xylol, and embedded in paraffin. Slices 5 μm thick with a Reichert-Jung microtome were made. Dewaxed slices were rinsed in PBS containing 0.5 M glycine, 1% serum albumin (BSA), and 5% goat serum albumin (GSA) and incubated for 2 h at room temperature in the primary antibody anti-αtubulin (Sigma), diluted 1:500 in PBS, 1%BSA, and 0.1%GSA. After rinsing in PBS, the secondary ab (FITC-conjugated anti-mouse immunoglobulin, Cappel, I) diluted 1: in PBS/BS/GSA was used for 2 h incubation in the dark. Specific controls were performed by omitting the primary antibody.

### Mathematical modeling of the exposure

The transport by diffusion of AT and CCh in the aqueous solution was modeled, to compute at each instant the exposure of the embryonic segments and to establish the related interference in the inductive events taking place in the single embryo structures at each developmental stage.

The general governing equation for the three-dimensional diffusion equation of a chemical substance, subject to a first order reaction in homogeneous phase and linear reversible and instantaneous sorption, can be written as:

1 $$R\frac{\partial c}{\partial t}={D}_{x}\frac{{\partial }^{2}c}{\partial {x}^{2}}+{D}_{y}\frac{{\partial }^{2}c}{\partial {y}^{2}}+{D}_{z}\frac{{\partial }^{2}c}{\partial {z}^{2}}-\lambda c$$

where *c* is the solute concentration [M cm^−3^] in the considered control volume, *x* is the longitudinal coordinate, *y* and *z* are the horizontal transverse and the vertical coordinates, respectively, *D*_*x*_ is the longitudinal diffusion coefficient [cm^2^s^−1^], *D*_*y*_ and *D*_*z*_ are the horizontal transverse and the vertical transverse diffusion coefficients [cm^2^s^−1^], respectively, *t* is time [s], *λ* is the first order decay constant [s^−1^], and *R* is the retardation factor [-] related to sorption.

The retardation factor can be eliminated from the term on the left by replacing *D*_*x*_, *D*_*y*_, *D*_*z*_ with *D*_*x*_/*R*, *D*_*y*_/*R*, *D*_*z*_/*R*, and *λ* with *λ/R*.

The initial condition can be expresses as follows:

2 $$c\left(x,y,z,0\right)=0$$

If we consider the source as a box of finite volume inserted into the domain, the following boundary conditions hold:

3 $$\begin{array}{ccc}c(\pm \infty ,y,z,t)=0& \mathrm{or}& \frac{\partial c(\pm {L}_{x},t)}{\partial x}=0\end{array}$$

4 $$\begin{array}{ccc}c(x,\pm \infty ,z,t)=0& \mathrm{or}& \frac{\partial c(\pm {L}_{y},t)}{\partial y}=0\end{array}$$

5 $$\begin{array}{ccc}c(x,y,\pm \infty ,t)=0& \mathrm{or}& \frac{\partial c(\pm {L}_{z},t)}{\partial z}=0\end{array}$$

where *L*_*x*_, *L*_*y*_, *L*_*z*_ [cm] are the borders of domain, supposed as finite or infinite, and the generation source term is expressed as:

$$r=\left\{\begin{array}{c}{r}_{0 }f\left(t\right) ; 0<x<{x}_{0}; {y}_{1}<y<{y}_{2} ; {z}_{1}<z<{z}_{2}\\ 0\end{array}\right.$$

The general solution of Eq. (1) in infinite domain and (3), (4), and (5) expressed as first type boundary conditions on concentration can be formulated as follows:

6 $$\begin{array}{c}c(x,y,z,t)=\frac{{r}_{0}}{8}{\int }_{0}^{t}f(t-\tau )exp\left[-\lambda (\tau )\right]\left[erfc\frac{x-{x}_{0}}{2\sqrt{{D}_{x}\tau }}-erfc\frac{x}{2\sqrt{{D}_{x}\tau }}\right]\\ \left[erfc\frac{y-{y}_{2}}{2\sqrt{{D}_{y}\tau }}-erfc\frac{y-{y}_{1}}{2\sqrt{{D}_{y}\tau }}\right]\left[erfc\frac{z-{z}_{2}}{2\sqrt{{D}_{z}\tau }}-erfc\frac{z-{z}_{1}}{2\sqrt{{D}_{z}\tau }}\right] \, {\text{d}}\tau \end{array}$$

This solution is valid for constant diffusion coefficients (Paladino et al. 2018).

To derive possible closed forms of this analytical solution, the boundary condition describing the source can be formulated as a Dirichlet (first type) or as a Newmann (second type) condition if a semi-finite *x* domain is considered. A Robin (third type) boundary condition cannot represent our experimental conditions since the velocity of the flux in input is negligible and anyway difficult to be evaluated (Massabo’ et al. 2011). With this approach, Eq. (3) can be reformulated as:

7 $$c(0,y,z,t)=\left\{\begin{array}{c}{c}_{0}f(t)\begin{array}{cc};& \end{array}-D\frac{\partial c(0,t)}{\partial x}=g(t)\\\ 0\end{array}\right.\begin{array}{c}\begin{array}{cc}-{L}_{y}<y<{L}_{y};& -{L}_{z}<z<{L}_{z}\end{array}\\ otherwise\end{array}$$

8 $$\begin{array}{ccc}c(+\infty ,y,z,t)=0& \mathrm{or}& \frac{\partial c(+\infty ,t)}{\partial x}=0\end{array}$$

where *c*_0_ [M cm^−3^] is the initial source concentration, *f*(*t*) is a dimensionless time function, and *g*(*t*) is a time function [M cm^−3^ s^−1^].

The main problem in modeling complex experiments involving drug release is the choice of reliable boundary conditions for the borders, i.e., well representing both the shape of the domain under study, and a good description of the source. To represent the experimental domain, the releasing source (the agar block) of dimension $2{L}_{x}* 2{L}_{y}* 2{L}_{z}$ is set with the releasing face at *z* = 0, so to have a plane source generating the drug that diffuses in a quasi-2D domain to the exposed cells of the embryo (Fig. 9 in Appendix). A semi-finite *x*-domain is considered, and the releasing agar reduces to a linear source. Regarding the lateral boundary conditions in *y*-domain, since the embryo is smaller than the diffusion domain (germinal chamber) and it is generally in the middle of it, the hypothesis of infinite boundaries holds completely true.

The analytical solution becomes:

9 $$c(x,y,z,t)=\frac{{C}_{0}x}{8\sqrt{\pi {D}_{x}}}{\int }_{0}^{t}f(t-\tau )\mathrm{exp}\left[-\lambda \tau -\frac{{(x)}^{2}}{4{D}_{x}\tau }\right]\left[erfc\frac{y-{L}_{y}}{2\sqrt{{D}_{y}\tau }}-erfc\frac{y+{L}_{y}}{2\sqrt{{D}_{y}\tau }}\right]\frac{1}{(\tau )}{\text{d}}\tau$$

Finally, as regards the description of the source release represented by $f\left(t\right)$, the agar is a semi-solid (colloidal) basal medium, and the mechanisms of drug release from it are complex, including the diffusion of the chemical into the colloid, its dissolution, the dissolution of the medium, swelling and erosion processes by solvents (Rivadeneira et al. 2018). In swellable releasing devices, the chemical is dispersed into a glassy hydrophilic polymer and then compressed to form a solid. A gel-like phase is formed due to water swelling, and the bioactive agent is released, usually with a first-order release kinetics (Brazel and Peppas 2000). In swelling-controlled release systems, the chemical is dispersed into the gel-phase as films, disks, or spheres, and it is released at the interface, corresponding to the water penetration front. In this case, relaxation of the hydrogel influences the diffusion mechanism of the water-soluble chemical that remains immobile and begins its diffusion as the polymer swells with water. So, at initial times there is a moderated release, then the continued swelling of the matrix causes the drug to diffuse increasingly easily, prolonging and linearizing the release curve. The release mechanism could be described by different kinetics, depending on the diffusion regime inside the agar.

In this simulation, we neglected the initial transient of drug release, whose characteristic time is much smaller than the diffusion (exposure) time. Moreover, since at the end of the experiments some residual drug was still present inside the agar, we put $f\left(t\right)$=1 in Eq. (7), so concentration at the boundary in *x* = 0 is constant, and equal to the average volumetric concentration of the chemical inside the agar, taken as *c*_0_, and computed by knowing both the volume of the agar and the quantity of chemical injected into it during the experiments.

Moreover, we considered an isotropous medium inside the germination chamber, so the diffusion coefficients in x and y direction are both equal to the drug diffusion coefficient in water. The considered values of $D$ are $6\pm 3*{10}^{-6}$ cm^2^ s^−1^ (Di Cagno et al. 2018). We must notice that the albumin surrounding the chick embryo is about 90% water and 10% proteins that are known for their strong drug binding activity. Somaratne et al. (2020) observed that egg white proteins can form a compact and microstructurally homogeneous gel at certain pH conditions, capable to reduce the diffusion coefficients of many chemicals. Conversely, the development of albumin-based drug delivery systems (Elsadek and Kratz 2012) suggests an opposite behavior for some particular drugs. The uncertainty here considered for the chosen diffusion coefficient is high, and could take into account these effects, even if they could be better described with a properly experimentally determined value of *D* in egg white. Another possible approach to consider the effect of albumin using the proposed model is to describe the bind as an adsorption mechanism and embed it into the retardation factor of Eq. (9), as proposed in (Peng et al. 2009), where an equilibrium reaction between the drug and the proteins is investigated.

Our proposed model is a deterministic physically based diffusion model, based on mass conservation principle. This means that the parameters inside the model have physical–chemical meaning, and they represent properties of the materials used in the experimental procedure. For this reason, it is not necessary to perform the model identification and the model validation steps to adopt it (Paladino et al. 2019). Anyway, even if parameters (i.e., the diffusion coefficient of the drugs inside the germination chamber) can be estimated in proper devoted diffusion experiments, in this case, the experimental punctual measurement of the concentration in time inside a germination chamber is not feasible (so synthetic egg white should be used). Therefore, model uncertainty exists; it is not due to the model shape, but it only derives from the errors in the chosen values of the diffusion coefficients, in this case estimated in water, and in the hypotheses embedded in the boundary conditions and describing the release mechanism.

### Microscope images and spatial dimensioning

The embryos stained in toto for AChE activity revelation (60 exposed to AT, 60 exposed to CCh and 40 exposed to physiological solution) were fixed, mounted in resin, and images were taken on the light microscope (Leitz, DE). The final position of each embryo with respect to the agar was observed and classified as in Fig. 1a. The embryo images were georeferenced into the domain space. To do this, both the distance from the source and the embryo length at the final time of exposure were experimentally measured.

Statistical elaboration of the measures was performed to establish if the variation of the dimensions was due to the exposure or to individual variability.

## Results

### Controls state and drug diffusion

The control samples exposed to physiological solution developed with a percentage of mortality and anomaly corresponding to the one present in the hatchery from which the eggs were obtained (about 8% during winter, 23% during hot summers). In both cases, the control samples were similar at morphological and histological analyses. Development of controls was rather synchronous, with individual differences in the range described by HH.

The experimental samples were exposed to the drugs at the same stage; the drugs reached the embryos with effective concentration at different times, depending on the distance and the relative position of the agar, so the development was not synchronous. The observed embryo position with respect to the agar was mainly distributed near to the cephalic and caudal portions of the body axis (70%). The drugs were continuously released by the agar, so waves of increasing concentration reached the developing structures during different morphogenetic events, according to the distance of the agar from the embryos.

To quantify the exposure, the spatial coordinates of the head, heart, and Hensen’s node inside the embryo were taken. The position of these structures was measured at the final time (embryo stage experimentally observed), so their position at the previous different times of development was extrapolated by estimating the growth velocity, supposed being linear (Fig. 1b).

#### Series EA of experiments: (3 groups of 60 eggs) for each experiment, repeated along the year

Exposure at 20 h incubation (T0, stage 4 H.H.), embryos collected at 50 h incubation (T30). Control embryo length at T0 = 1.04 ± 0.2 mm; cephalic process and node position at a mean distance of 0.2 mm; control embryo length at T30 (stage 14–15 H.H) = 5 ± 0.5 mm; mean head length = 1.4 mm; node position at the end of the body. The mean growth velocity during the experiment was 0.13 ± 0.03 mm/h. (Fig. 1b). In controls, the presumptive position of the hind part of the head and future position of the heart was at 0.2 mm from the top of the embryo at stage 5 (T0) and at 1.4 mm ± 0.02 mm at stage 13 (T30).

#### Series EB of experiments: (3 groups of 60 eggs) for each experiment, repeated along the year

Exposure at 24 h incubation (stage 6 H.H.), embryos collected at 33 h incubation (stage 9–10).

Control embryo length at final sampling time = 2.9 ± 0.5 mm. The mean calculated growth during the experiment was 0.15 ± 0.03 mm/h. Presumptive position of the hind part of the head and heart position were at 2/5 the embryo length (Fig. 1b).

### Effect of drug concentration at different developmental stages

Concentration profiles in time were then generated by the model for each relevant position (Supplementary Material, Online Resource Table S1).

**Fig.2:**
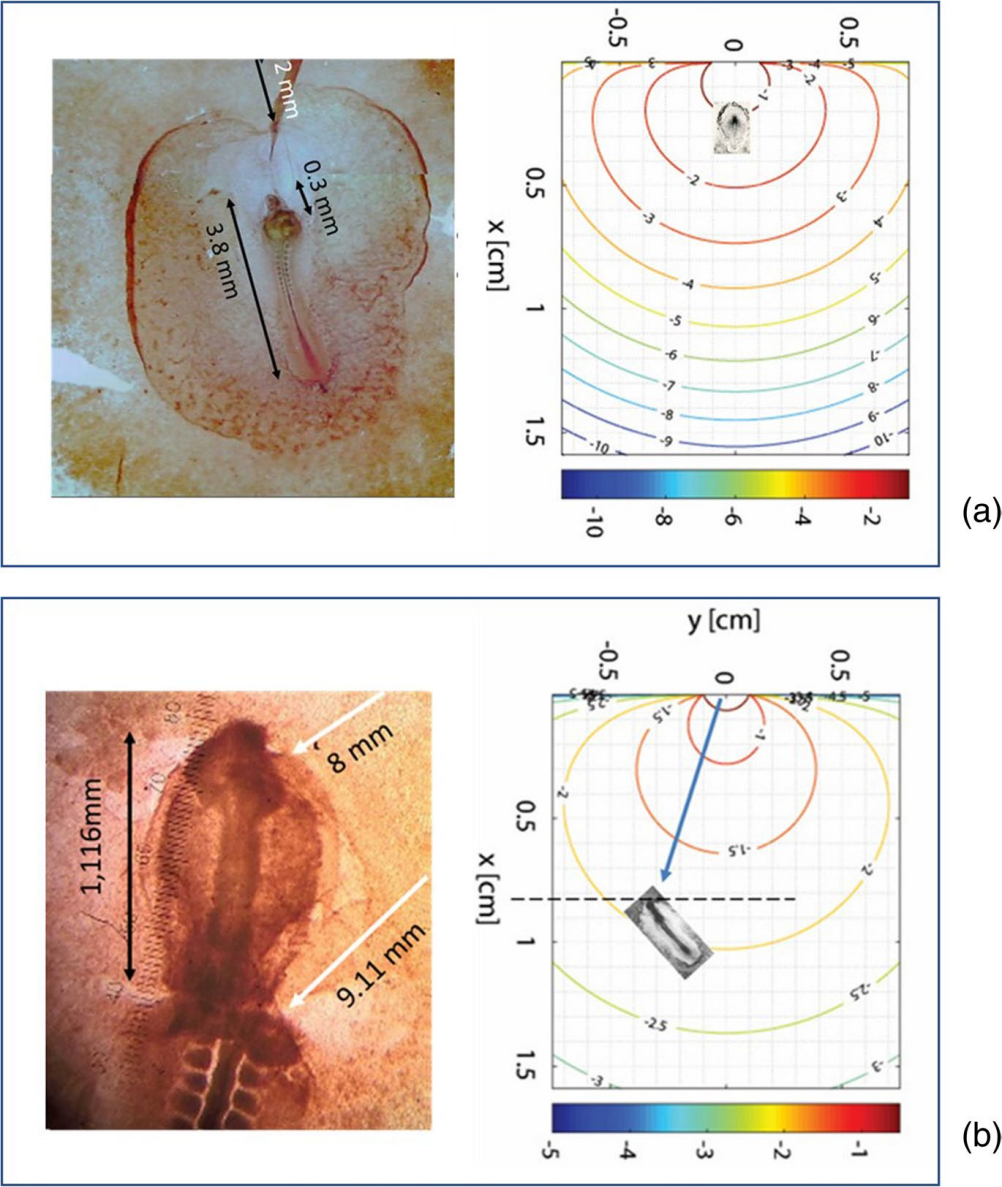
Embryos exposed to agar soaked with 3 µL of 10^−3^ M AT. **a** Cephalic position, agar 2 mm far from the head, collected at T30 (stage of control 14–15 HH). Strongly anomalous head and heart: 10 somite pairs (compatible with stage 10 HH). Brown-magenta staining of active AChE localized in the neural tube and heart of the embryo, somites, and final primitive streak. In the area opaca, AChE reaction is mainly present in the peripheral vein and in the blood islets of the caudal half, i.e., opposite to agar. Not well-defined area pellucida. **b** Cephalic-lateral position, the white arrows show the distance of the agar from the main structures of the body. Each square in the field represents 1 mm.^2^

The effects on embryos development depended on different parameters:

1. Distance from the source. At constant *T* (time from exposure), concentration decreased from the point 0 (source) along the field (Table S1);
2. Time needed to reach the developing structures. At constant *d*_*s*_ (distance from the source) concentration increased with time, proportionally from T0 to T30 (Fig. 10 in Appendix; Table S1);
3. Embryo stages. The effects of exposure were influenced by the stage of the embryos, as the most severe anomalies occurred when the effective concentrations reached the embryos at earlier stages (Fig. 2). When the concentrations capable of interfering with development reached the structures of the embryo at late stages, only the differentiating structures were affected by the exposure.

When the agar is near to the embryo, the effective concentrations are reached in less than 3 h (10^−4^ M for AT, 10^−3^ M for CCh) while the cephalic process and the prechordal plaque are forming. These in control samples differentiate to the whole cranial basis and will induce the formation of the cephalic CNS. Generally, head malformation is accompanied by malformation or failure to form the heart, which is located far from it according to the stage and individual variability.

Figure 2a shows the effects of agar soaked with AT, in cephalic position, 2 mm far from the embryo. Exposure to atropine at T2-T4 (respectively 1.09 × 10^−4^ ± 1.4 × 10^−5^ M and 1.268 × 10^−4^ ± 1.45 × 10^−5^ M) during the first 3 h prevented the elongation of the cephalic process and the formation of the prechordal plaque, so that the head was not formed. In this case, the effective drug wave was extended at time T8 to the presumptive site of heart formation, with estimated concentration 1.18 × 10^−4^ ± 1.06 × 10^−5^ M, causing anomalous folding of the vessels. The average concentration reaching the hind part of the body (5.92 × 10^−5^ ± 6.6 × 10^−6^ M) was not able to cause visible anomalies, except a delay in development and growth.

In addition, the model allowed to calculate for each differentiating structure, the difference between the effective and non-effective concentration.

Figure 2b represents an embryo sampled at T30 (stage 12–13 in controls) at apparent stage 10. The source of AT was 8.2 mm from the head, left at the top (arrow). The left optical vesicle is scarcely differentiated. The differentiation of optical vesicles begins between stage 8 and stage 9 HH that is approximately 8 h after the start of the experiment. At this time, the concentration of AT on the left vesicle was about 1.0288 × 10^−5^ M, while on the other vesicle, distant 8.4 mm fron the agar and moved to the right side by 0.3 mm, the concentration according to the calculation in S1 was 0.912 × 10^−5^ M. The difference between the two vesicles was 0.1168 × 10^−5^ M. Thus, the minimum effective concentration (LOEC) for the development of the eye turns out to be about 1.0288 × 10^− 5^ M, and the deviation of 0.117 × 10^−5^ M makes the difference. The hind parts of the body therefore do not suffer effects, except a slowdown in development and a lesser activity of AChE in the left opaque area, not quantified in the image.

### General effects of AT and CCh

#### Exposure to AT

##### Series EA of experiments

**Fig. 3:**
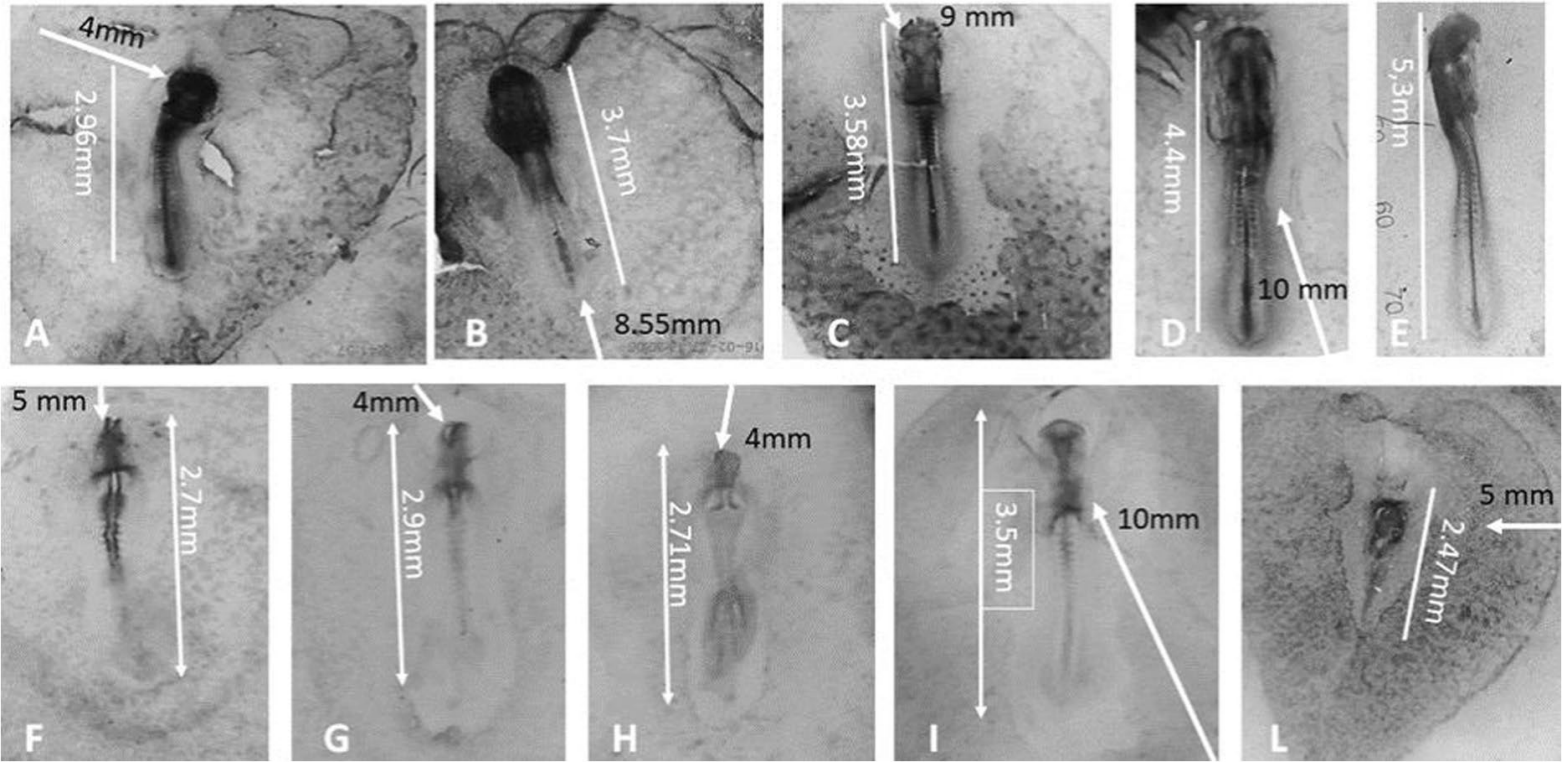
Embryos exposed to agar soaked with atropine. A–D, exposure starting from stage 4 (T0 = 20 h incubation) and collected at stage 14 (T30 = 50 h total incubation). E, control. F–L, embryos exposed since 24 h (stage 6, average length 1.5 mm) and collected at 33 h, corresponding to stage 10. The arrows indicate the distance between the agar and the initial position of the Hensen’s node. I, control. L, dead embryo

10^−3^ M AT (agar close to the embryo) was the most effecting concentration, causing disruption of the embryogenesis in 50% cases; the concentration 10^−4^ M caused effects in 35% cases; the concentration 10^−5^ M caused effects in differentiating structures in 20% cases, generally slowing down the development and the number of somites (Fig. 3). During the stages investigated, 10^−6^ M caused mild effects or nothing.

##### Series EB of experiments

At T6, the prechordal plaque is already formed, so the head organization is not affected, as well as the average length of the embryos sampled at T9 (2.98 ± 0.18 mm). AT exposure generally delayed the development of the single structures at the time when the inductive messages took place. In Fig. 3(F, G, H), we can observe the effects of a drug concentration between 2.5 × 10^−5^ and 8.07 × 10^−6^ M, reaching the embryo head after 2–3 h from the beginning of the experiment (T7-T8), while the embryos were at stages between 7 and 8 (HH). This exposure impaired the head development, as well as the neural fold closure. Figure 3(I) shows the effect of the drug released from a distance of 1 cm: at 3 h it reached the head at 3.05 × 10^−7^ M, allowing the formation of the eye cups, but since concentration increased with time, the drug reached the vitelline veins at T8 at concentration 5.56 × 10^−6^ M, impairing in this case their fusion to form the heart. Nevertheless, the length of the embryo and the somite pairs’ number is compatible with stage 9–11 (T8, 33 h incubation, according to Fig. 1b).

#### Exposure to CCh

**Fig. 4:**
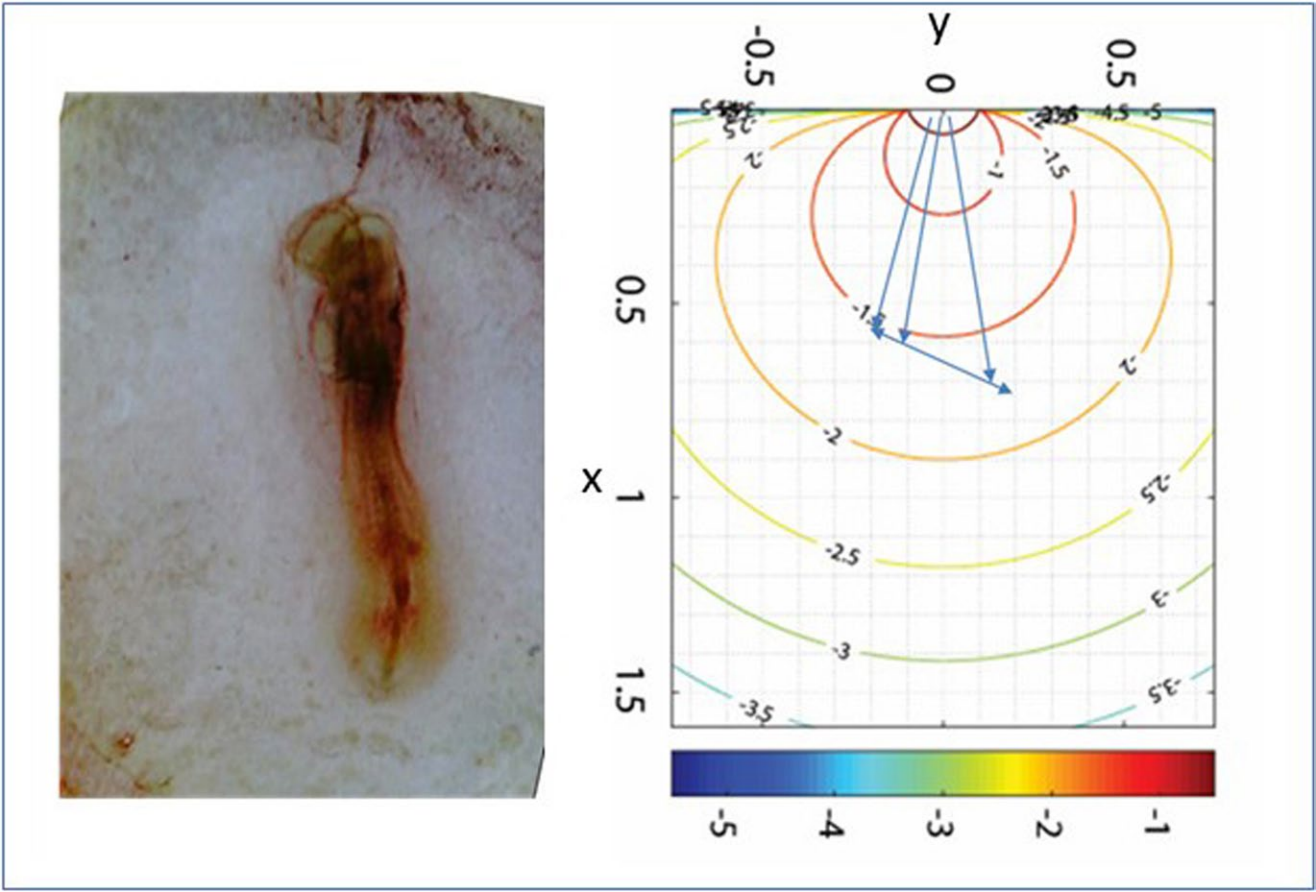
Embryo exposed to CCh at T0 = 20 h, in cephalic-lateral position, sampled at T = 50 h. Initial carbachol concentration in the agar: 10^−1^ M; direction of the flux: cephalic lateral. The eyes are not formed, the head is not closed anteriorly (corresponding to cephalocele condition) the heart is tubular, but not well formed. The primitive streak residual is short and thick.

Exposure to CCh high concentration at early stages was able to disrupt the body plan, by preventing the closure of the neural tube. Figure 4 shows the effect of the agar 5 mm far, in cephalic-lateral position. Final embryo length: 3.8 mm; head length = 1.26 mm: distance agar-head = 5.1 mm: agar-heart = 6 mm; agar-tail = 7.6 mm. The drug concentration at the single districts varied with time: during the closure of the cephalic neural folds, at 7 h development, the average concentration on the head structures was 3.65 × 10^−3^ ± 3.73 × 10^−5^ M. At T8, during the initial heart differentiation, the average concentration along the presumptive area was 2.58 × 10^−3^ ± 3.41 × 10^−5^ M. The whole embryo was 3.8 mm long, and the number of visible somites was compatible with stage 8–9 HH (T10 in our experiment). Average concentration of CCh from the heart to the tail = 2.6 × 10^−3^ ± 3.44 × 10^−5^ M.

**Fig. 5:**
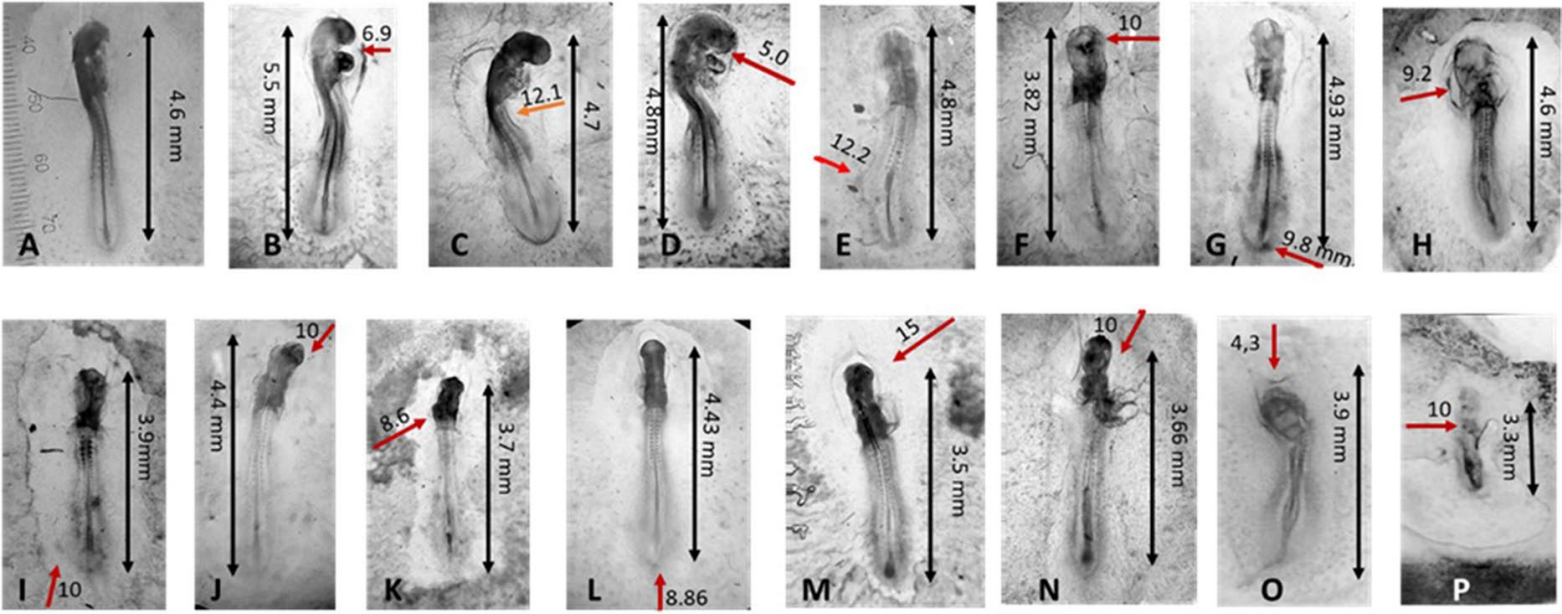
A selection of typical anomalies of embryos exposed to agar soaked with 10^−1^ M carbachol. The red arrows show the direction and distance (mm) of the agar from the embryo. A: control, B–P: exposed embryos. The portions of the embryo directly exposed to the flux show enhanced growth and scarce differentiation (B, C, D, N: head and heart; AChE activity, shown by a dark precipitation is also enhance or irregularly distributed (56.52% embryos). O, P: lethal anomalies due to the near position of the agar (6% embryos). A general trend (43.47% of anomalous embryos) is represented by neural tube partially closed or lowered respect to the controls (e.g.: E, F, H, I, K, N). The percentages refer to anomalous embryos, which are less than 25% of the total treated items

In Fig. 5, a panel of pictures shows the effects of exposure to CCh at different distances and positions of the agar with respect to the embryos. The greatest effect of CCh was exerted on the rhythm of cell division, which caused a growth of the structures at the expense of their differentiation. In Fig. 5, the arrows show the direction of the drug flux towards the embryos. The difference of the effect is visible on structures a few millimeters apart from each other, though the concentration of the drug varies very little (as calculated by the model). When the source of CCh is located caudal, the growth in length of the embryo is visible, with little differentiation of head and heart, which remained small and elongated (Fig. 5G-L). For example, in Fig. 5L the CCh concentration at the end of the tail is 1.08 × 10^−3^ M, while at the head it is 4.48 × 10^−4^ M, with a difference of 6.32 × 10^−4^ M. When the source is lateral, the most directly exposed part appears more developed towards the drug wave, compared to the rest of the body. (e.g., Fig. 5J, K). In this case (Fig. 5K), the right eye cup as well as the right branchial pouch seem more developed than the left ones. According to Table S1, the CCh concentration on the left part of the body was 1.17 × 10^−3^ M, while on the right side, it was 9.65 × 10^−4^ M, with a difference of 2.05 × 10^−4^ M.

#### Comparison of exposure to AT and CCh

**Fig. 6:**
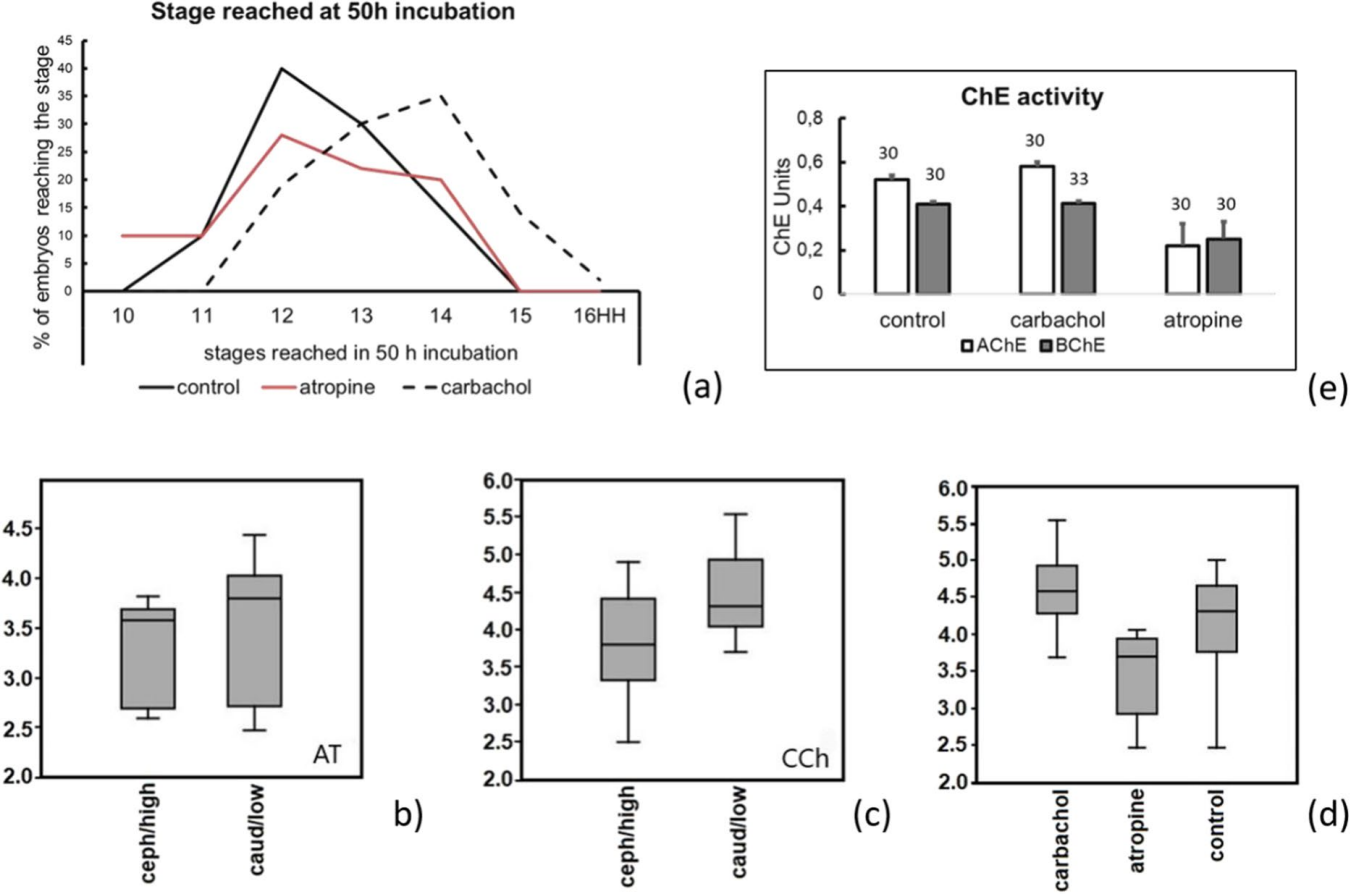
Comparison of embryos exposed to the cholinomimetic drugs AT and CCh; agar about 1 cm far from the blastodisc. **a** Stages of development reached, numbered according to HH. **b**, **c** Length of the embryos according to the position of the agar: ceph/high = agar in cephalic or lateral high position, caud/low = agar in caudal or lateral low position, *Y* axis = embryo length [mm]. **b** Exposure to 10^−5^ M AT, *P* > 0.01. **c** Exposure to 10^−3^ M CCh, *P* < 0.008. **d** Dimension of the embryos, *Y* axis = embryo length [mm]: AT vs control *P* < 0.001; AT vs CCh *P* < 0.001; control vs CCh: *P* > 0.05 (Kruskal–Wallis). **e** Expression of AChE activity in whole embryo homogenates

Development was stage-dependently accelerated or decelerated according to the exposure to AT or CCh, independently from the concentration of the drugs: while atropine lowered, exposure to CCh generally enhanced the speed of development as compared with controls (Fig. 6a). The position-related effects of the drugs gradient seemed to be independent from the direction of the gradient (*P* > 0.01) for embryos exposed to AT (Fig. 6b), while they seemed to depend on the agar position for embryos exposed to CCh (Fig. 6c). The general trend of AT exposure was to cause smaller embryos, while CCh caused embryos bigger than controls (Fig. 6d).

### AChE activity localization and measure

AChE activity, shown by the dark brown-magenta precipitation in all the pictures, was mainly affected by AT and generally depressed in the whole body of the embryos, including the embryonic annexes, such as area opaca and vitelline veins, except in the organs strongly damaged, where the activity was strong and deeply stained (Fig. 3, Fig. 4). In the area opaca and its blood islets (when these were present), most of the residual activity was present in the vascular area opposite to the agar, while it was not present in the portion of area opaca corresponding to the direction of the drug’s flux (Fig. 3A, B, and C).

CCh exposure generally slightly enhanced the activity of the enzyme, as resumed in Fig. 6e.

### Effects of muscarinic inhibition on the CNS architecture

**Fig. 7:**
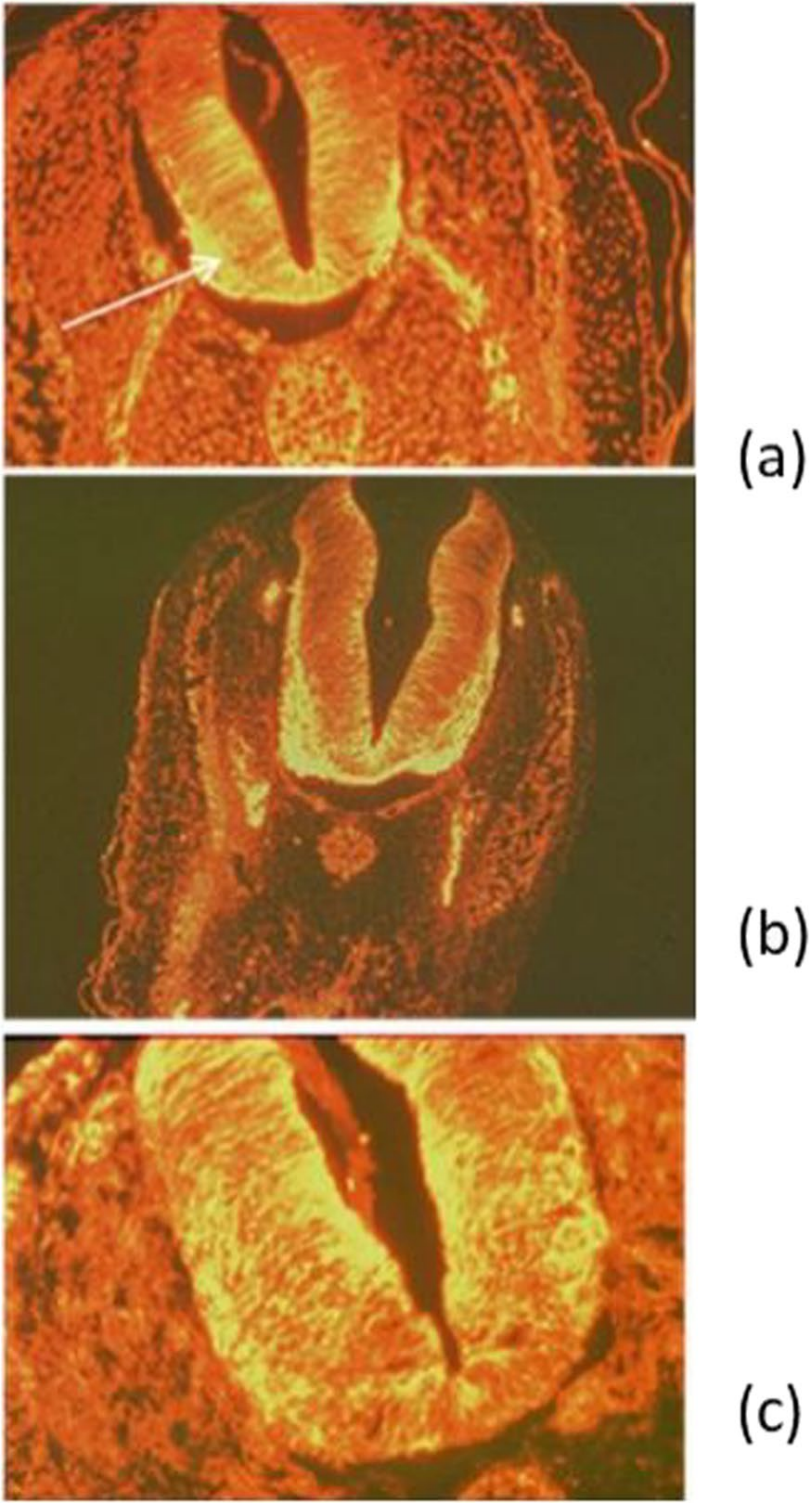
Embryos sampled at T30. Sections (3 μm thick). IF revelation of α-tubulins in the motor neurons and fibers. a Control embryo. b Embryo exposed to 10^−3^ M ACh. c Embryo exposed to 10^−4^ M atropine, agar cephalic, 1 cm far. The figure shows deficient differentiation of the interested structures in the embryos exposed to 10^−4^ M AT, as compared to control and ACh exposed embryos

**Fig. 8:**
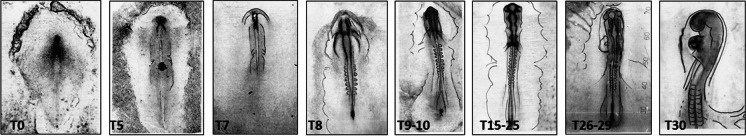
Stages from 20 h (T0, stage 4 HH) to 50 h incubation (T30); AChE histochemical staining in toto; the reaction products stain with a dark precipitation the positive sites. The photos were retouched in pen to highlight the main organs and tissues. T5 = St7 HH, 2 somite ps; T7 = St.7 HH, 3–4 somite ps; T9-10 = St.8 HH, 4–8 somite ps; T13 = St 9–10 HH;T13-15 = St.11 HH, 10–13 som ps; T25-29 = St. 13 HH, 16–18 somite ps;T 30 = St.14–15 HH, 19 somite ps

Figure 7 shows the effect of exposure to 10^−4^ M AT since the stage 5 HH on the differentiation of the ventral motor neuron architecture, as compared to control and ACh exposed chick embryos. As for CCh, acetylcholine (ACh) does not affect differentiation, while AT impairs the specification of the motor portions of the neural tube.

## Discussion

For the exposure to cholinomimetic substances, our model follows the behavior of morphogens, which are released from a localized source, forming a concentration gradient over a population of nearby and distant cells that respond directly to the signaling molecule in a concentration-dependent way (Gurdon and Bourillot 2001).

The results show that the effect of the muscarinic drugs is exerted on embryo development depending mainly on the time of exposure, and the effect impinges on the events occurring at that specific time of development. As earlier, the exposure occurs to effective concentrations, as more dramatic is the effect. This is because development is a multiphasic event, where each phase originates from the former, thus amplifying the effects along time (Donnelly and Corman 2008). In these events, AChRs regulate a wide variety of physiological responses, including apoptosis, cellular proliferation, and neuronal differentiation (Resende and Adhikari 2009). MAChRs are known to exert an effect on the eye differentiation and dimensions, as reported by Angelini et al. (1998). The fact that CCh causes an increase in embryo size matches with the report that AChR activation is involved in the regulation of the rate of cell proliferation (Resende and Adhikari 2009). In fact, it has been shown that in the early stages of embryonic development, the excitation of muscarinic receptors mediated by ACh or CCh enhances while AT inhibits the intracellular release of calcium ions, associated with the nuclear breakdown preceding the early cleavages (Harrison et al. 2003). The major effects anyway were exerted on the anterior part of the embryo, independently from the position of the agar. This may be due to the exposure time used in this work, which was long as compared to the hemi life of AT inside adult organisms (approximately from 2 to 4 h). Thus, the differentiation of caudal structures may be less dramatically affected for this cause or because the morphogenetic fields are restricted and already committed to their fate. The identification of the chord mesoderm position depends on the expression of a maternal gene, *siamois* (Kohn and Moon 2005), which is expressed very early, before the beginning of the experiments here described. As seen in Fig. 2a, the formation of the notochord and of the overlying neural tube proceeds caudally, independently of the presence of the cephalic structures, which can be removed without altering this event (Healy et al. 2001). In addition, when the agar is in cephalic position, the cephalic part maintains about the same distance from the agar, while the most caudal part moves away. Thus, the effect of AT / CCh on the final length of the embryo may be due to a slower/accelerated mitotic index, rather than to interference in the inductive messages.

Overall, the effects of muscarinic blockade were generally milder than the effects of other cholinomimetic substances such as cholinomimetic pesticides (Falugi et al. 2011) as the action of AT is mainly directed to muscarinic receptors, so that its effects are milder than the one of other anti-cholinergic drugs with mixed effect (Sakharova et al. 1995).

According to Freeman and Gurdon (2002), development may be seen as a continuing series of cell interactions that guide cells and tissues progressively towards their fate. Development depends on the interaction of ligands (morphogens) and their receptors, which have as their answer the expression of appropriate genes. These are activated at different times by a clock mechanism or by inputs from the environment. In this case the environment is represented by the surrounding cells and tissues. In this complex network of ligands and specific receptors, acetylcholine receptors play a role in regulating the pathway of intracellular responses by modulation of intracellular calcium ions. Thus, the mAChRs blockade/activation may interfere with the expression of the genes involved in each event (Table 1).

**Table 1:** Pleiotropic interference of muscarinic drugs in different inductive events

Exposure time	Developmental events	Inductive interaction	10^−4^ M AT int	10^−3^ M CCh int
T0	Cephalic process, body axis position	Autonomous, maternal *siamois* expression (Kohn and Moon 2005)	+	No
T4	prechordal plate	Signal from notochord to endoderm. (Chan et al. 2001),	+	No
T7	cephalic fold	Signal from prechordal plate and notochord to ectoderm (Healy et al. 2001)	+	No
T10	neural tube closure (neural crest migration)	Cytoskeletal movements (tubulin/actin)	+	+
T10	Vascular area	Mesodermal induction to endoderm	+	+
T13	Optic vesicles	Pax genes, inductive messages from neural tube to ectoderm and vice versa (Kozmik 2005)	+	+
T13	Brain pattern specification	Otx, Emx homeobox genes (Cecchi et al. 2000)	+	No
T10-T13	Architecture of neural tube basal plate (motor neurons)	Induction by contact from notochord to neurectoderm (Placzek et al. 1993)	+	No
T13-T15	Heart formation	Hedgehog and RAS pathways (Liu et al. 2006)	+	No

T0-T13 body axis and body plan establishment. T16-T30 the morphogenetic fields and potentials are restricted to the single body parts shaping and growth. *Int* interference

During these events, a complex series of interactions between morphogens and their receptors is taking place along gradients of ligands (morphogens) expression. The cells recognize different threshold concentrations of morphogens through receptors on their surface and transduce this information to the nucleus which in turn expresses the correct genes driving a specific differentiation (Gurdon and Bourillot 2001). According to our outcomes, AT exerted an opposition to the morphogen reception and intracellular conduction, while CCh enhanced the morphogen signaling, and this interference caused errors of morphology expression.

**Fig. 9:**
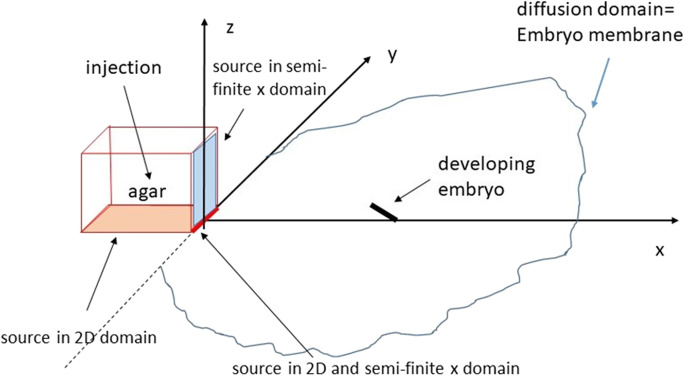
Scheme of drugs diffusion, 3D view

**Fig. 10:**
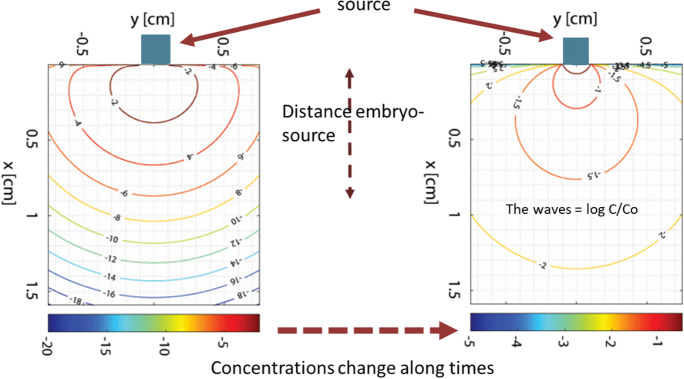
graphical representation of the diffusion field of the drugs, from the source (S). The numbers on the waves represent the log C/C_0_ (concentration/concentration at the source). Left: diffusion field at 1 h exposure; right: diffusion field at 30 h exposure. Each square represents 1 mm.

The mathematical model has shown that a small difference in concentration within the same embryo is sufficient to have different responses in the different districts, located at a very small distance from each other. A small concentration difference is sufficient both for AT (Fig. 5) and for CCh (Fig. 9 in Appendix) to pass from inhibition of the differentiation to simple slowing down or even no-effect.

## Conclusions

Muscarinic drugs do not act as morphogens, but interfere with the pathways of different morphogens, by altering the intracellular concentration of Ca2 + ions, which in turn can interfere with the intracellular traffic of organelles and molecules (including regulatory proteins) linked to the cytoskeletal dynamics (as it was demonstrated in sea urchin embryos by Aluigi et al. (2008).

The mathematical model was the only way to establish the effects of inhibition/activation of muscarinic receptors in individual events: dose, time, and event dependence at the same time.

### Electronic supplementary material

Below is the link to the electronic supplementary material.

Supplementary file1 (DOCX 12 KB)

Supplementary file2 (XLSX 9892 KB)

## Data Availability

The authors declare that all the data supporting the findings of this study are available within the article and its supplementary information files. Other microscope photos are available from the corresponding author on reasonable request.

## Abbreviations

Ab: Antibody
ACh: Acetylcholine
AChE: Acetylcholinesterase (E.C. 3.1.1.7)
AChRs: Acetylcholine receptors
AcTChI: Acetylthiocholine iodide
AT: Atropine
BChE: Butyrylcholinesterase (E.C. 3.1.1.8)
BTChI: Butyrylthiocholine iodide
CCH: Carbachol, carbamylcholine
HH: Hamburger and Hamilton, 1951
IF: Immunofluorescence
PFA: Paraformaldehyde
TS: Physiological Tyrode solution

## Code availability

The codes to solve the mathematical model of diffusion generated during the current study (Matlab codes) are available from the corresponding author on reasonable request.
