# Supplementary material for: Spatiotemporal role of muscarinic signaling in early chick development: exposure to cholinomimetic agents by a mathematical model
Source: Cell Biol Toxicol. 2022 Sep 13;39(4):1453–69. doi: 10.1007/s10565-022-09770-w (PMC10425487; doi:10.1007/s10565-022-09770-w)
Supplement: Supplementary file 1 — Supplementary file1 (DOCX 12 KB) [file 10565_2022_9770_MOESM1_ESM.docx]

Figure S1: Interactive measurement of the drug concentrations. The single pages of the excel file present the concentration of the drug at different times, identified by the coordinates of the cartesian plane showing the position of the embryo and of its parts. Pages (at the basis) show the incubation hours; X, Y= distance from the source and embryo position in the plane.  The found concentrations should be multiplied for the initial concentration of atropine and carbachol (10^-3^ and 10^-1^M, respectively).
